# Regulatory mechanisms of mitochondrial BK_Ca_ channels

**DOI:** 10.1080/19336950.2021.1919463

**Published:** 2021-05-06

**Authors:** Ana L. González-Cota, Carmen Santana-Calvo, Rocío Servín-Vences, Gerardo Orta, Enrique Balderas

**Affiliations:** a Departamento de Genética del Desarrollo y Fisiología Molecular, Instituto de Biotecnología, UNAM. Av. Universidad 2001, Cuernavaca, Morelos, México; b Instituto Gulbenkian de Ciência. Rua da Quinta Grande 6, Oeiras, Portugal; c Instituto de Tecnologia Química e Biológica, Universida de Nova de Lisboa. Av. da República, Oeiras, Portugal; d Department of Neuroscience, The Scripps Research Institute. 10550 North Torrey Pines Road, La Jolla, CA, USA; e Nora Eccles Harrison Cardiovascular Research & Training Institute, University of Utah, Salt Lake City, UT, USA

**Keywords:** Mitochondria, genetic origin, DEC sequence, BK_ca_ channels, Maxi-K channels, beta subunits, amyloid beta

## Abstract

The mitochondrial BK_Ca_ channel (mitoBK_Ca_) is a splice variant of plasma membrane BK_Ca_ (Maxi-K, BK_Ca_, Slo1, K_Ca_1.1). While a high-resolution structure of mitoBK_Ca_ is not available yet, functional and structural studies of the plasma membrane BK_Ca_ have provided important clues on the gating of the channel by voltage and Ca^2+^, as well as the interaction with auxiliary subunits. To date, we know that the control of expression of mitoBK_Ca_, targeting and voltage-sensitivity strongly depends on its association with its regulatory β1-subunit, which overall participate in the control of mitochondrial Ca^2+^-overload in cardiac myocytes. Moreover, novel regulatory mechanisms of mitoBK_Ca_ such as β-subunits and amyloid-β have recently been proposed. However, major basic questions including how the regulatory BK_Ca_-β1-subunit reaches mitochondria and the mechanism through which amyloid-β impairs mitoBK_Ca_ channel function remain to be addressed.

## Introduction

Mitochondrial ion channels play key roles in maintaining and regulating mitochondrial volume, biogenesis, membrane potential, Ca^2+^ homeostasis, metabolism and a broad spectrum of physiological processes derived from mitochondrial physiology including cell death. Amongst other mitochondrial ion channels so far described, mitoBK_Ca_ channel received special attention since its pharmacological activation exerts cardioprotection, as early noted by the O’Rourke laboratory in 2002 [1]. The genetic ablation of BK_Ca_ in cardiac myocytes expanded these early observations and revealed that mitoBK_Ca_ is a splice variant of plasma membrane BK_Ca_, and that its targeting to the inner mitochondrial membrane (IMM) strongly depends on a short stretch of amino acids located at the C-terminal (the DEC sequence) [2]. The molecular recognition of the mitoBK_Ca_-DEC segment by members of the mitochondrial outer membrane import system (TOM) assures proper target of the channel into the IMM [3]. Moreover, recent findings have revealed that the association of mitoBK_Ca_ with auxiliary BK-β1 subunit regulates its expression, targeting, and shifts the channel’s voltage sensitivity, which has important physiological consequences on mitochondrial Ca^2+^ handling [4]. Novel mechanisms of regulation of mitoBK_Ca_ channels such as amyloid-β (Aβ) are also emerging. Aβ is a peptide that accumulates in brain in Alzheimer´s Disease and inhibits the activity of mitoBK_Ca_ channels [5]. Confirmation of this effect by other laboratories might have important implications in our understanding of AD and possibly on the development of new treatments for neurodegenerative diseases. In this review, we will expand our discussion of the basic properties of mitoBK_Ca_ contained in the most recent papers. We will start discussing the evolution and conservation of the mitochondrial targeting sequence (DEC) amongst vertebrates and the most recently discovered mechanisms that control the expression, targeting and activation of this channel in mitochondria. In the last part of this review, we discuss the novel modes of regulation of mitoBK_Ca_ channels and its fundamental roles in mitochondrial and cell physiology.

### The molecular nature of mitoBK_Ca_

**Figure 1.**
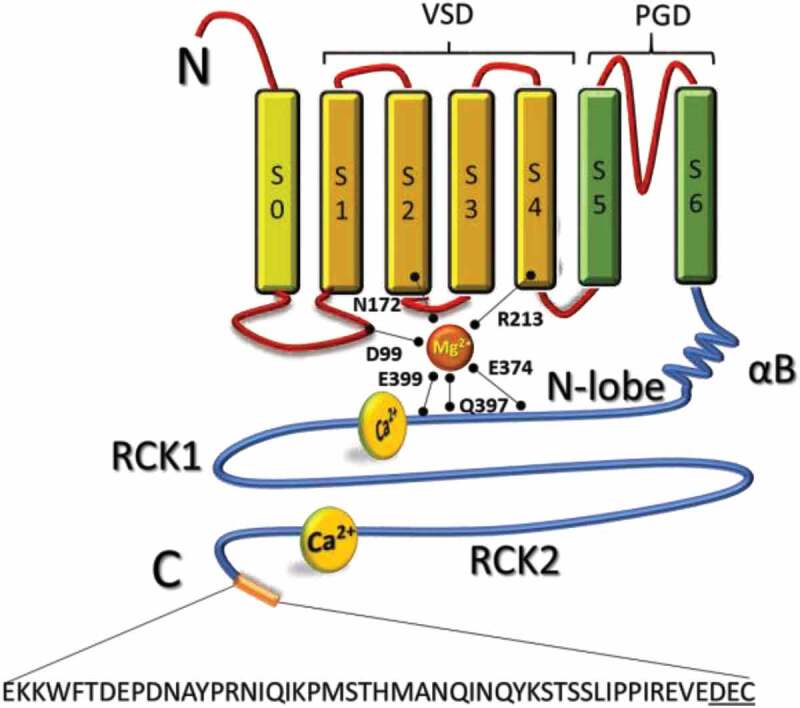
Structural components of the mitoBK_Ca_ channel α subunit. Schematic topology of mitoBK_Ca_ α subunit. The α-subunit is composed by 7 transmembrane domains (S0-S6) and N-terminal (cytoplasmic) and C-terminal (matrix) opposites. The S1-S4 domains constitute the voltage sensing domain (VSD) and the S5-S6 domain conform the pore gating domain (PGD). The C-terminal domain (CTD) is connected to the transmembrane domain through an alpha helix/beta-sheet linker (αB), each connecting S6 to the rest of the N-lobe of the Regulator of Potassium Conductance (RCK) 1 domain (residues 344–613). The “gating-ring” contains residues D99; N172; R213; E374; Q397 and E399 important for activation of the channel by Mg^2+^; and a second RCK domain (residues 718–1056). High affinity Ca^2+^-binding sites located at RCK 1 and 2 conform the “Ca^2+^-bowl”. At the end of the CTD a 50 amino acid insert contains the DEC sequence specific to target mitoBK_Ca_ channel. Four α subunits form a functional channel

In rodent cardiomyocytes, mitoBK_Ca_ is a splice variant of the plasma membrane BK_Ca_, *KCNMA1* [2]. Unlike the canonical mitochondrial targeting relying on signal peptides at the N-terminal, the mitoBK_Ca_ possesses a 50 amino acid sequence at the C-terminal denominated as “DEC” after the last three amino acids (Figure 1) that promotes the import of the channel to the IMM [2].

### The genetic origin of mitoBK_Ca_-DEC sequence

**Figure 2.**
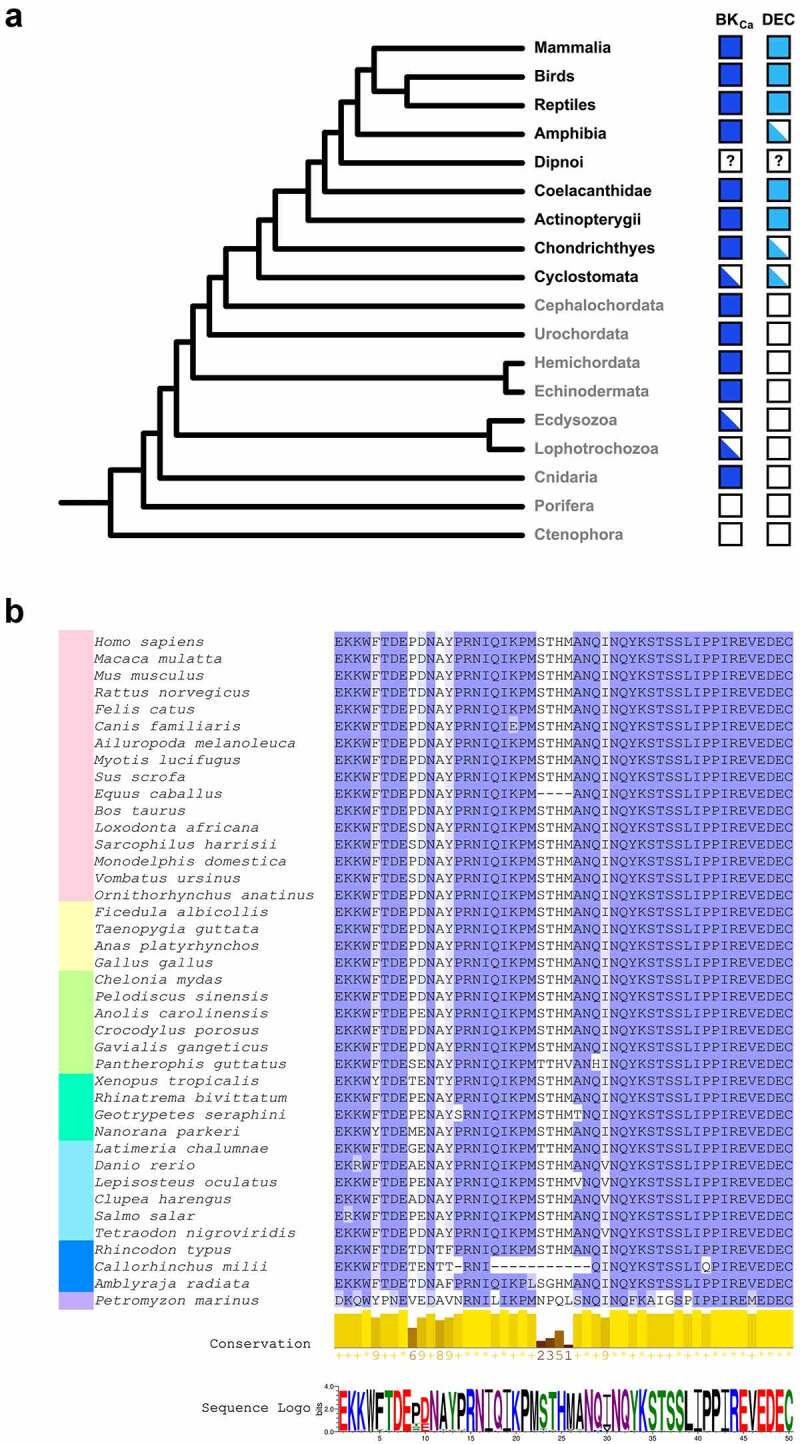
Conservation of BK_Ca_-DEC sequence. (**A**) A simplified metazoan phylogeny is represented describing the presence or absence of BK_Ca_ and DEC sequences in the examined taxa. Taxonomic groups belonging to vertebrates and invertebrates are depicted in black and gray font, respectively. The tree topology was done by phyloT based on the NCBI taxonomy and visualized in the interactive Tree of Life tool (https://itol.embl.de/itol.cgi). Solid color boxes indicate that the correspondent sequence was identified in all the organisms searched for that taxonomic group. Half-filled boxes indicate that at least one, but not all, of the organisms belonging to that taxonomic group possess the sequence. A white box represents the cases where the sequence was not found; in the case of dipnoi we cannot assure complete absence since only genomic traces and transcriptomic data were available. The search of BK_Ca_ (KCNMA1) and its DEC sequence were done in NCBI database, using their respective *Homo sapiens* sequence as initial query for BLASTp and tBLASTx. Additional rounds of BLAST searches were performed using hit sequences from the first round to identify potential distantly related homologs that might not be detected by using the initial query sequences. To corroborate that the sequences obtained belonged to bona fide BK_Ca_ channels a search for the Ca^2+^ bowl sequence [6] and the GYG (or GxGD) pore motif [71] was performed *posteriori*. (**B**) Alignment of DEC sequences found in vertebrates. The colored bar at left indicates the taxonomic group, pink for mammals, yellow for birds, green for reptiles, turquoise for amphibians, light blue for bony fishes (actinopterygians plus coelacanths), dark blue for Chondrichthyes (cartilaginous fishes) and purple for cyclostomes (jawless fish)

To understand the evolutionary relationship between the BK_Ca_ and the DEC sequence we performed a BLASTp and tBLASTx search for both sequences in the NCBI database (https://www.ncbi.nlm.nih.gov). Interestingly, the hits for the DEC sequence were solely present in proteins annotated as BK_Ca_ or SLO-like channels, suggesting a novel and probably unique mechanism for mitochondrial import of these proteins. To determine if the hits obtained for the BK_Ca_ belonged to *bona fide* BK_Ca_ channels we searched for the Ca^2+^ bowl sequence and the highly conserved GYG (or GxGD) pore motif. For our surprise, while *bona fide* BK_Ca_ channels are widely conserved throughout the animal kingdom (Figure 2A), the DEC sequence is only present in vertebrates showing a high degree of conservation among this group (Figure 2B), suggesting a conserved mechanism for mitochondrial targeting.

Despite these results, the activity of mitoBK_Ca_ channels has also been observed in invertebrates such as *Caenorhabditis elegans* [7] and *Drosophila melanogaster* [8], both lacking the DEC sequence, suggesting the existence of additional mitochondrial targeting mechanisms in those taxonomic groups. In addition, BK_Ca_-like currents have been observed in planar lipid bilayers reconstituted of mitochondrial membrane fractions from the potato *Solanum tuberosum* [9], and the protist *Dictyostelium discoideum* [10]. While the molecular identity of BK_Ca_ was assigned based on western blots with the use of an anti-K_Ca_1.1 antibody [9,10], our blast search shows that neither *S. tuberosum* nor *D. discoideum* possess a *bona fide* BK_Ca_ channel encoded in their genome, neither the epitope sequence for the anti-K_Ca_1.1 is present in their sequences. Hence, more experiments including loss of function and knockout mutants need to be performed to elucidate the molecular identity responsible for these currents in these evolutionary distant organisms.

The presence of the DEC sequence in all the groups of vertebrates might indicate that a channel with characteristics such as large conductance for K^+^ and exquisitely regulated by Ca^2+^, plays an important role in mitochondrial physiology, that once selected during evolution has suffered minor changes in the subsequent younger taxa. Yet, our understanding of the precise role(s) that mitoBK_Ca_ plays in such evolutionary distant organisms and in multiple organs and cell types in the same organism has just begun.

### Structure of BK_Ca_ and mitoBK_Ca_

As a splice variant of the plasma membrane BK_Ca_, the overall structure of mitoBK_Ca_ might be conserved. The basic architecture of the pore forming α-subunit can be divided in a transmembrane domain consisting of seven transmembrane segments (S0-S6) and a cytoplasmic domain (Figure 1), while three major structural domains can be recognized: i) a voltage-sensor domain formed by charged residues located at the S2, S3 and S4 segments [11,12,13,14]; ii) a pore-gate domain (S5-S6) through which K^+^ ions are conducted; and iii) a cytoplasmic domain that contains Mg^2+^ binding sites and the regulators for conductance of K^+^ or RCK domains that bind Ca^2+^. Four α-subunits encoded by the *KCNMA1* gene form a functional mitoBK_Ca_ channel (Figure 1) and four pairs of RCKs (RCK1-RCK2) form the Ca^2+^-sensing apparatus, the so-called “gating-ring” occupying two-thirds of the whole BK_Ca_ structure.

### Biophysical properties of mitoBK_Ca_

Over the past 20 years, a large collection of papers has described the most fundamental biophysical properties of mitoBK_Ca_ channel (Table 1). Most of the work has been performed using the patch-clamp technique applied to mitoplasts (inner mitochondrial membrane devoid of outer mitochondrial membrane). With this technique, Siemen and coworkers were the first to establish that mitoBK_Ca_ channel from a glioma cell-line LN229 has large conductance for K^+^ as well as a voltage and Ca^2+^ sensitivity [15], like its counterpart at the plasma membrane [16,17,18]. A pioneer assessment of a physiological role of mitoBK_Ca_ in cardioprotection, was done by the O’Rourke laboratory revealing that cardiac mitoBK_Ca_ was sensitive to changes in matrix Ca^2+^ and could be blocked by charybdotoxin (ChTx) applied to the external face of the channel [1]. Moreover, pharmacological activation of mitoBK_Ca_ with NS1619 proved to be cardioprotective reducing the infarct size of hearts treated with this mitoBK_Ca_ opener [1]. These early studies did not only shape the pharmacological profile of mitoBK_Ca_ channel but also represented a milestone in the understanding of the physiological role of BK_Ca_ in mitochondrial and cell physiology. The studies that followed these seminal works revealed that the conductance slope of mitoBK_Ca_ varies among different tissues and cell types [19,20]. Moreover, the unitary conductance of cardiac mitoBK_Ca_ has proven to be diverse (Table 1), ranging between 190 and 300 pS [1,21,22]; in a recent report [23], a cardiac mitoBK_Ca_ channel from mice displayed a conductance of 145 pS in 150 mM symmetric KCl and 100 µM Ca^2+^. This variability suggests the existence of heterogeneous conductances for K^+^ in cardiac mitochondria, as recently demonstrated by 22, where a conductance of 190 pS was assigned to mitoBK_Ca_, among other conductances observed in the same preparation of mitoplasts, those ranging between 60 and 370 pS [22]. We had recently found that cardiac mitoBK_Ca_ channel has a conductance ranging between 290 and 320 pS under symmetric 150 mM KGluconate and 10 µM matrix Ca^2+^ [4]. It is possible that different ionic conditions employed in other studies caused such variability in the reported conductances for mitoBK_Ca_.

**Table 1.** Biophysical and pharmacological properties of mitoBK_Ca_ channels

Cell Type	Methods (Patch Clamp)	Conductance (pS)	Associated Subunit	Inhibitor*	References
**Human glioma cell line (LN229)**	Mitoplast	295	ND	ChTX [1.4 nM)	15
**Guinea pig ventricular myocytes**	Mitoplast	307	ND	ChTX (200 nM], IbTX [100 nM)	1
**Rat brain**	Mitochondria lipid bilayers	260–320	ND	ND	72
**Rat ventricular myocytes**	Mitoplast	270	β1	Paxilline [3 µM]	21
**Human glioma cell line (LN229 and LN405]**	Mitoplast	276	ND	ChTX (100 nM)	73
**Rat astrocytes**	Mitoplast	295	ND	IbTX (100 nM)	74
**Rat brain**	Mitochondria lipid bilayers	265	β4	ChTX (200 nM), IbTX (50 nM)	75,76
**Potato (*Solanum tuberosum*) tuber**	Mitochondria lipid bilayers	502–615	β2	IbTX [200, 400, 600 nM)	9
**Rat brain**	Mitochondria lipid bilayers	211	ND	IbTX (100 nM], TEA (10 mM), 4-AP [10 mM)	81
**Rat brain**	Mitochondria lipid bilayers	565	ND	IbTX (100 nM], ChTX (200 nM), 4-AP [10 mM)	81
**Human endothelial cells line (EA. Hy926]**	Mitoplast	270	β2	IbTX (100 nM), Paxilline (10 µM)	82
**Mouse cardiac myocytes**	Mitoplast	190	ND	Paxilline [100 nM)	22
**Mouse ventricular myocytes**	Mitoplast	145	ND	Paxilline [10 µM]	23
**Rat ventricular myocytes**	Mitoplast	300	β1	Paxilline [100 nM]	4

**Note**: *Concentrations used in the cited studies.

**Abbreviations**: ND, No determined; ChTX, charybdotoxin; IbTX, iberiotoxin; TEA, tetra-ethyl ammonium; 4-AP, 4-aminopyridine.

### Activation of BK_Ca_ channel

Intracellular Ca^2+^ concentration ([Ca^2+^]_i_) and membrane depolarization can allosterically activate plasma membrane BK_Ca_ channel [17,24,25,26]. Likewise, Ca^2+^ and membrane potential exquisitely activate mitoBK_Ca_. Since mitoBK_Ca_ is encoded by the same gene, KCNMA1, we can expect structural and functional conservation. In this regard, BK_Ca_ channel open probability increases as a function of Ca^2+^ concentration and, as has been described for mammalian Slo1 channels, the Ca^2+^-activation curve is a function of membrane voltage [24].

### Voltage activation of BK_Ca_ channel and Ca^2+^ binding to the gating ring

It is well established that BK_Ca_ channel gating is also regulated by voltage, involving charged amino acids located in S2-S4 transmembrane segments [11,12,13,14,27,28,29,30,31,32,33,]. Allosteric interactions between Ca^2+^ or voltage sensors can open the channel independently as well as synergistically, enabling the channel to functionally couple intracellular Ca^2+^ signals with the electrical activity of the cell [34]. The intracellular gating ring of eukaryotic BK_Ca_ channel comprises eight RCK domains (Figure 1). Each BK_Ca_ channel subunit contains two nonidentical RCK domains (RCK1 and RCK2) linked in tandem [35], thus forming an intracellular gating ring of four RCK1–RCK2 tandems.

### Arrangements on the RCK domain transduce in the opening of BK_Ca_

The structural changes that occur after binding of Ca^2+^ to the RCK domains have provided important clues to understanding the channel opening. Experimental studies pioneered by the Olcese laboratory [36,37], revealed conformational changes of the RCK1 and RCK2 domains induced by Ca^2+^, as well as an elegant optical demonstration that Ca^2+^ binding to the intracellular BK regions allosterically facilitates the activation of the voltage sensing apparatus of the channel [38]. Moreover, the recently determined crystal structure of Slo1 from *Aplysia californica* revealed that binding to the Ca^2+^ bowl and RCK1 sites in the C-terminal domain (CTD) leads to a near rigid-body lateral tilting (away from pore) of the N-lobes formed by the upper part of each RCK1 domain (Figure 1) [39,40,41]. This lateral tilting moves the RCK1 attachment point for each S6-RCK1 C-linker laterally and downward, pulling on S6 to potentially open the pore gate. Simultaneously, the lateral tilting of the N-lobe moves the αB helix located at the top of each N-lobe (Figure 1) both upward and laterally to push on the bottom of the S4–S5 linker/VSD to potentially open the channel. In addition, it has recently been demonstrated that in human BK_Ca_, the αB helix links the binding of Ca^2+^ at the RCK domains to the VGD of the channel, confirming that interaction between the αB helices at the top of the N-lobes of the CTD and the cytoplasmic surfaces of the S4-S5 linkers/VSD is required to open the channel [42]. In agreement with this new model for channel opening, early observations have shown that both RCK1 and 2 domains can move independently from each other upon binding of Ca^2+^, indicating a high degree of flexibility for this domain [43]. Moreover, Giraldez and Rothberg expanded these observations establishing that ligand binding to the RCK domains stabilized the active conformation of the BK_Ca_ channel [44]. Despite these important advances, questions regarding the activation of mitoBK_Ca_ by voltage and Ca^2+^ remain open. In excitable cells, large non-physiological amounts of Ca^2+^ are required to activate the BK_Ca_ channel. When expressed alone the BK_Ca_ α subunit shows a voltage of half activation (V_1/2_) of 18 mV at 10 µM [Ca^2+^]_i_, which is shifted toward negative and relatively physiological values (V_1/2_ = −77 mV) when co-expressed with its regulatory β1 subunit [45]. Strikingly, in cardiac mitochondria one population of mitoBK_Ca_ channels showed a V_1/2_ = −55 mV at 12 µM [Ca^2+^]_i_, indicative of functional association with auxiliary β1 subunits [4]. Shifting the voltage sensitivity of mitoBK_Ca_ by association with regulatory subunits and its allosteric activation by elevating mitochondrial matrix Ca^2+^, helps to define the physiological window where opening the channel might occur. In this context, largely hyperpolarized mitochondrial membrane potential (ΔΨ ~-200 mV) might keep the channel mostly closed, maintaining the large driving force for Ca^2+^ and the physiological processes that depend on it. On the other hand, under stress conditions such as ischemia, opening of this large conductance for K^+^ has proven to be cardioprotective when treated with NS1619, a BK_Ca_ opener. Moreover, opening of mitoBK_Ca_ correlates with a higher capacity of mitochondria to handle Ca^2+^ [1,2,4]. Although proximity with the sarcoplasmic reticulum (SR) ensures mitochondrial Ca^2+^ uptake, rises in matrix Ca^2+^ must be tightly controlled particularly under high stress conditions such as ischemia and/or metabolic dysfunction. Thus, opening of mitoBK_Ca_ might depolarize mitochondria reducing the driving force for Ca^2+^, thus preventing the initiation of apoptosis and cell death. We will discuss the experimental evidence that support this hypothesis in the following sections.

### Expression of mitoBK_Ca_ channels in adult cardiomyocytes

Adult rodent cardiomyocytes express a splice variant of plasma membrane BK_Ca_. This splice variant localizes exclusively at the IMM and chemical activation of cardiac mitoBK_Ca_ channel reduces the infarct size after ischemic insult [1]. Moreover, hearts from the BK-KO (*KCNMA1*^−/-^) treated with the BK_Ca_ opener NS1619 did not show this protection against ischemic insult [2]. The study by Singh and coworkers elegantly demonstrates the importance of mitoBK_Ca_ channel in cardiac function; however, the mechanism(s) through which mitoBK_Ca_ prevents cardiac damage remains to be fully elucidated.

### The physiological role of mitoBK_Ca_ channel in controlling Ca^2+^ overload

To understand the role that mitoBK_Ca_ channel might play in protecting cardiac tissue after an ischemic insult, it is necessary to review the function of plasma membrane BK_Ca_ channel in other cell systems. The rhythm of vital physiological processes depends on the dynamics of Ca^2+^ entry and membrane potential, both triggers of BK_Ca_ activity. Action potentials (AP) in neurons and smooth muscle cells depend on the activation of voltage-dependent calcium channels (VDCC) which in turn increase cytosolic Ca^2+^ [46] and activate neighboring BK_Ca_ channels [47,83,84]. This functional coupling causes a massive K^+^ efflux through BK_Ca_ channels that rapidly repolarizes (<1 ms) the membrane potential by shutting down the VDCCs, shaping and ensuring propagation of the AP. In smooth muscle cells, activation of BK_Ca_ channels has a negative-feedback effect in contractility by reducing entry of Ca^2+^ via the VDCCs [48]. A similar feedback mechanism might occur in cardiac myocytes, where Ca^2+^ and K^+^ play major roles in contraction-relaxation processes. During cardiac AP, a depolarization of the plasma membrane activates VDCC. Influx of Ca^2+^ induces the release of more Ca^2+^ from the SR through the activation of the ryanodine receptors (RyR). This rapid elevation of cytosolic Ca^2+^ ensures activation of the myofilaments contracting the myocytes. The delicate balance between contraction and relaxation largely depends on the rapid extrusion/removal of Ca^2+^ from the cytoplasm, which occurs mainly through the active recapture of Ca^2+^ into the SR via the Ca^2+^-ATPase (SERCA) and through extrusion of Ca^2+^ via the plasmalemmal Na^+^/Ca^2+^ exchanger (NCX). Detachment of Ca^2+^ from its binding sites on the troponins lead to the relaxation of myocytes. This dynamic and perfectly coordinated mechanism commonly known as cardiac excitation-contraction coupling (ECC) accounts for the proper pumping of 6000 l of blood per day in the adult human heart. Adult cardiomyocytes express a large battery of K^+^ channels responsible to restore the membrane potential that terminates the cardiac AP [49]. Intriguingly, the large conductance for K^+^, voltage-dependent and Ca^2+^-activated BK_Ca_ channels do not take part in this process mostly due to their exclusive expression in mitochondria [2]. It has been previously hypothesized, and excellently reviewed by [50], that the activation of a large conductance for K^+^ might help to modulate mitochondrial Ca^2+^ overload, a critical step preventing mitochondrial permeability transition pore (mPTP) opening and cell death. In metazoans, mitochondrial Ca^2+^ uptake occurs mainly through the mitochondrial calcium uniporter (MCU) [51], that uses the large driving force for Ca^2+^ established by the activity of the electron transport chain [52]. Rise in the mitochondrial matrix Ca^2+^ would activate the mitoBK_Ca_ channel [15], which in turn would depolarize the mitochondrial membrane potential reducing the mitochondrial Ca^2+^ driving force [53], preventing the mitochondrial Ca^2+^ overload, and thus the formation and opening of the mPTP [21,54,55,56]. In agreement with this hypothesis, we observed that blocking the mitoBK_Ca_ channel with paxilline impaired the ability of mitochondria to control Ca^2+^ overload. Our observations also indicate that mitoBK_Ca_ is functionally associated with auxiliary subunits of the β1-type [4]. To understand the physiological role of this association, we must briefly review the well-documented modes of regulation exerted by BK-auxiliary subunits on plasma membrane BK_Ca_.

### Association of plasma membrane BK_Ca_ with auxiliary subunits

Expression of plasma membrane BK_Ca_ pore forming α-subunit is commonly accompanied by the expression of auxiliary β (1–4), γ (1–4), or both types of subunits. Auxiliary BK_Ca_-subunits β and γ (mostly tissue-specific) modify the kinetics of the channel, Ca^2+^ and voltage sensitivities, and toxin sensitivity [see 57,for a detailed review on this topic]. Moreover, auxiliary β subunits can also act as modulators of channel density at the plasma membrane and mitochondria via endocytic processes [4,19].

### *Auxiliary BK_Ca_-β-subunits (KCNMB1*–*4)*

BK_Ca_-β subunits possess two transmembrane domains (T1 and T2) connected by an extracellular loop, with both N-terminal and C-terminal domains located cytosolically. As mentioned earlier, association of BK_Ca_-α subunit with auxiliary β1 subunits at high [Ca^2+^]_i_ (10 µM), shifts the V_1/2_ of activation from 18 mV to −77 mV relative to the expression of the α-subunit alone [45]. This functional association also prevents inactivation and rectification of the channel [58]. In addition, association of BK_Ca_-α subunit with auxiliary β2, and β3 subunits (comprising four splicing variants, a-d, in humans and primates) mediate fast inactivation and instantaneous current rectification [59,60]. Association with BK_Ca_-β2 affects the movements and equilibrium of the S3-S4 region, promoting opening of the channel by favoring the activated state of the voltage-sensor [61]. Overall, the auxiliary β2 subunits shifts BK_Ca_ V_1/2_ toward more negative membrane potentials, ranging from 27 mV [61] to 75 mV shift at 3–4 µM [Ca^2+^]_i_ [62]. However, the most distinctive characteristic of β2-containing BK_Ca_ channels is their fast inactivation [58,63].

### Regulatory BK_Ca_-γ-subunits

Auxiliary γ subunits belong to the Leucine-Rich Repeat (LRR) superfamily. The four γ subunits have an N-terminal signal peptide, an extracellular LRRC domain with an N-terminal cysteine-rich segment (LRR-NT), six LRRs, and a C-terminal cysteine-rich segment (LRR-CT), a single transmembrane domain, and a short cytosolic C-terminal tail [64]. Each γ subunit has a unique tissue-specific expression pattern and modulates the BK_Ca_ voltage dependence in heterologous expression systems [65]. Of the identified γ subunits, only γ1 has been established as a definitive BK_Ca_ channel regulator in native cells. This subunit shifts the V_1/2_ of BK_Ca_ from 168 to 10 mV in the absence of Ca^2+^ [66], and from 31 to −85 mV at 10 µM free [Ca^2+^]_i_ [67]. Auxiliary γ1 subunit also induces resistance to mallotoxin [68]. Homotetramers of BK_Ca_ channels can accommodate up to four γ1 subunits, one γ1 per α-subunit; however, a single γ1 is sufficient to produce the full gating shift of the channel [67,69,70]. As explained before, functional association of BK_Ca_ channel with its regulatory subunits modulates their biophysical properties, and ensures proper targeting and activation of the channel, crucial steps for cellular excitability, maintenance of Ca^2+^ homeostasis, triggering of signaling cascades, neurotransmitter release, among other physiological processes [29,71,77,78]. It is known that in the cardiovascular system, the expression and association of plasma membrane BK_Ca_ channels with auxiliary β1 subunit regulates vascular tone and blood pressure [79,80]. Moreover, this association is also preserved in mitochondria from adult cardiomyocytes [4]. Nevertheless, to date the association of mitoBK_Ca_ channel with auxiliary γ-subunits remains unknown.

### The expression, targeting and activity of mitoBK_Ca_ depends on its association with regulatory β1 subunits

We recently observed that the capacity of cardiac mitochondria to handle Ca^2+^ is linked to the expression of the BK_Ca_-β1 subunit. Mitochondria from β1-KO mice show a reduced capacity to retain Ca^2+^ and early opening of the mPTP, which correlates with lower expression and low P_o_ of mitoBK_Ca_ channel [4]. These results indicate that regulatory β1-subunit controls the activity of mitoBK_Ca_ and consequently mitochondrial Ca^2+^ handling. Despite the evidence, it is hard to reconcile the activation of mitoBK_Ca_ channel in the context of mitochondrial physiology (ΔΨ ~-200 mV, [Ca^2+^]_mit_ < 200 nM), where mitoBK_Ca_ channel must remain closed. Despite this, it is worth noticing that mitoBK_Ca_ displays a remarkable high P_o_ at negative membrane potentials in different cell types [1,15,81]. As noted, mitoBK_Ca_ displays a hyperpolarized V_1/2_ of activation relative to that of the BK_Ca_-α subunit when it is expressed alone [45,85,86,87,88,89]. Opening of the channel at hyperpolarized membrane potentials is enhanced upon its association with its regulatory β1 subunit [4,21,90]. Yet, the mechanisms by which the auxiliary BK_Ca_-β-subunits are targeted to the mitochondria and the roles that these subunits might play in health and disease remain to be determined. A new line of evidence suggests that regulatory β1 subunits might participate in the translation of mechanical stimuli into gating of BK_Ca_ channels [91]; however, as we will discuss in the next section, the mechanosensitivity of BK_Ca_ channels has yet to be fully determined.

### Additional regulatory mechanisms of mitoBK_Ca_ channels

It has been recently published that a subpopulation of mitoBK_Ca_ channels is modulated by mechanical stimulation. The authors found a slight increment in the P_o_ of mitoBK_Ca_ channels from human astrocytoma cells in response to mechanical stimulation (from 0.016 at 0 mmHg to 0.3 at −40 mmHg at +20 mV) [92]. Inherent mechanosensitive ion channels have evolved to detect and transduce mechanical forces into electrical signals, evoking substantial changes in their P_o_ in response to a mechanical stimulus. Amongst them, bacterial channels MscL and MscS [93,94], and eukaryotic channels PIEZO1, TRAAK and TREK1, as well as the most recently described members of the OSCA family [95,96,97]. Voltage-dependent and Ca^2+^-activated BK_Ca_ channels sense and gate the pore in response to changes in both membrane potential and elevation in cytosolic [Ca^2+^], increasing their P_o_ [11,12,13,14,17,24,25,26,29,30,31,27,28,32,33,98]. Those extensively studied mechanisms of activation strongly contrast with a rather negligible change in the P_o_ displayed by BK_Ca_ in response to large mechanical stimulus. Even more, when compared to changes in P_o_ observed in well-characterized mechanosensitive ion channels such as PIEZO1 and 2 [99, 100], the assessment that BK_Ca_ channels are capable to respond to a mechanical stimulus must be carefully revised. Despite the efforts to identify a structural domain on BK_Ca_ channel that acts as a “mechanosensor,” this remains as an open question. A study conducted by Zhao and Sokabe suggested the presence of a mechanosensitive domain in BK_Ca_, which is the linker that connects the transmembrane segment S6 with the RCK1 domain [101]. Moreover, shortening the linker results in an increased membrane-stretch sensitivity, whereas the opposite effect was observed by lengthening the linker, suggesting this site as the sensor of membrane tension. Paradoxically, the mutant with the longer linker also showed a reduced voltage and calcium sensitivity, indicating that the mutation of this region (S6) might affect the overall function of the channel. In addition, the stress-regulated exon (STREX), which is a cysteine-rich domain (CRD) located between RCK1 and RCK2 domains in the STREX-BK_Ca_ splice variant, has been proposed to be an essential element for the stretch sensitivity of BK_Ca_ channel [102]. STREX anchors the C-terminal of the BK_Ca_ to the plasma membrane [102,103] by a palmitoylation modification throughout the cysteine residues C12 and C13 within the CRD [103,104]. It is worth noting that the C-terminal from ZERO-BK_Ca_ (BK_Ca_ channel without the STREX insert) remains in the cytoplasmic side [103] and ZERO-BK_Ca_ alone does not respond to mechanical stimulus as shown by 102. Furthermore, single amino acid substitution from Ala674 to Thr674 within the STREX (ERA sequence) on BK_Ca_ channels completely abolished the stretch sensitivity [102]. An interaction of STREX with the cytoskeleton that ultimately may translate into a slight opening of the channel could not be ruled out. Further experiments are required for BK_Ca_ channels to be considered as mechanosensitive, together with a mechanism of activation and a physiological meaning for this property, thus far this phenomenon should not be considered as an inherent property of BK_Ca_ channels.

In the study by 92,the mRNA containing the STREX exon was detected in human astrocytoma cells. However, the splice variants of mitoBK_Ca_ containing the DEC sequence alone or together with STREX were not detected. Thus, it is not clear if mitoBK_Ca_ channels from human astrocytoma cells contain the STREX exon and whether the expression of this splice variant is sensitive to mechanical stimuli. Complementary research would help to assess mitoBK_Ca_ as an inherent mechanosensitive channel, including but not limited to channel reconstitution in proteoliposomes and stretch application through the patch-clamp pipette, together with loss or gain of function assays that might depend on the expression of different splice variants such as STREX.

### Modulation of mitoBK_Ca_ by amyloid-β (Aβ)

Alzheimer’s disease (AD) is the most common neurodegenerative disease characterized by neuronal loss, progressive cognitive deterioration associated with the reduction of daily activities and behavioral changes in elder people. The aggregation of Aβ peptides in the human brain has a neurotoxic effect and plays a key role in the development of AD [reviewed at 105]. Aβ is a self-aggregating peptide produced by the cleavage of a transmembrane glycoprotein, the amyloid precursor protein. In addition, mitochondrial dysfunction [see 106,for a review in this topic] and Ca^2+^ unbalance are among the most prominent hallmarks of AD [reviewed at 107]. In neurons, Aβ peptides promote Ca^2+^ release from endoplasmic reticulum (ER) increasing intracellular Ca^2+^ levels [108]. Neighbor mitochondria take up this Ca^2+^ inducing loss of mitochondrial membrane potential, generating ROS, and leading to apoptosis and cell death [108]. In addition, Aβ peptides can affect directly mitochondrial physiology since they accumulate in mitochondria [106,109]. Studies in vitro have shown that Aβ peptides are imported through the TOM complex and predominantly localized at the IMM [109]. Interestingly, a recent study on mitoplasts from human astrocytoma cells has reported that different forms of Aβ, including monomers, oligomers and fibrils inhibit the activity of mitoBK_Ca_ channels in a concentration dependent manner. The highest concentration of Aβ fibrils tested (5 µM) produced an 80% inhibition, whereas Aβ monomers and oligomers inhibited 50% and 70% of mitoBK_Ca_ channel activity, respectively [5]. All forms of Aβ inhibited mitoBK_Ca_ channel activity when applied at either side of the membrane [5], indicative of an indirect effect on the channel. As it has been reported that Aβ oligomers modify the tension of the plasma membrane and disrupts the cytoskeleton [110], Kravenska and coworkers proposed that Aβ forms induce a mechanical change that transduces into closure of mitoBK_Ca_ channel [5]. As we stated in a previous section of this review, the opening of mitoBK_Ca_ correlates with a higher capacity of mitochondria to handle Ca^2+^ in cardiomyocytes [1,2,4]. If this is the case in neurons, we can hypothesize that the presence of Aβ in the IMM might contribute to the development of AD through the inhibition of mitoBK_Ca_, which ultimately could lead to mitochondrial damage and cell death. Thus, it becomes relevant to confirm these findings in proper AD models to determine the molecular mechanisms and signaling pathways through which Aβ affects the biophysical properties of mitoBK_Ca_ channel and how this affects mitochondrial and neuronal physiology. This will improve our understanding of AD development and perhaps would help us to design specific strategies to prevent or treat AD.

## Concluding remarks

Evidence about the constitution, regulation, origin, and evolution of the mitochondrial BK_Ca_ channel is emerging. More importantly, it has been shown that for a successful targeting of mitoBK_Ca_ to the mitochondria needs to bear the DEC sequence and, in some tissues, interact with the auxiliary subunit β1. Here we showed that the DEC sequence is solely associated with BK_Ca_ channels, and it is highly conserved and exclusively present in vertebrates. Although these findings might contribute to our understanding of the physiological role of mitoBK_Ca_ in the organisms that bear the DEC sequence, their full significance remains to be clarified. Moreover, new questions arise about the mechanisms of mitochondrial targeting; especially considering that large conductances for K^+^ have been described as mitoBK_Ca_ channels in organisms that do not contain the DEC sequence. Regulation of mitoBK_Ca_ channels by auxiliary β1-subunit and amyloid-β peptides was recently proposed. Although most of the questions remain open, the modulation of mitoBK_Ca_ through these mechanisms could be of high relevance in the development of pathophysiological conditions such as ischemia and neurological diseases where mitochondria have a crucial role. Thus, the study of the signaling pathways and the molecules implied in regulation of mitoBK_Ca_ might contribute to the understanding and future treatment or prevention of these conditions.
